# Supplementary material for: Complexome profiling of the Chlamydomonas psb28 mutant reveals TEF5 as an early PSII assembly factor
Source: Plant Cell. 2025 Mar 17;37(6):koaf055. doi: 10.1093/plcell/koaf055 (PMC12164590; doi:10.1093/plcell/koaf055)
Supplement: koaf055_Supplementary_Data [file koaf055_supplementary_data.zip › psb28_interactive.zip!/plots/intensity/qualityPlots/peptides/ycf4_Cre-1.g2717009.t1.p1.html]

### Cre-1.g2717009.t1.p1

Trivial name: ycf4  
SeqCoverage [median]: 23.90  
Mass: 22.42 kDa  
  
 

| Sequence | MissedCleavage | Mass [Da] | wtIntensity | muIntensity | Score |
| --- | --- | --- | --- | --- | --- |
| DIEAIRVELK | 1 | 1184.68 | 0.00 | 5356.06 | 53.76 |
| EIPLTGIGQPLTLK | 0 | 1478.87 | 21082.74 | 14423.61 | 142.97 |
| QGLDAQR | 0 | 786.40 | 7854.03 | 12306.66 | 127.12 |
| TQNNILIR | 0 | 970.56 | 24878.07 | 46192.91 | 225.57 |
| TQNNILIRR | 1 | 1126.66 | 763.81 | 856.48 | 64.71 |
| VELKQGLDAQR | 1 | 1255.69 | 283.29 | 0.00 | 53.57 |
| YIIVGER | 0 | 848.48 | 441.95 | 14245.57 | 99.38 |
|  |  |  | 55303.9 | 93381.3 |  |
